# 2-(4-Nitro­benzyl­idene)malononitrile

**DOI:** 10.1107/S1600536812008896

**Published:** 2012-03-03

**Authors:** Ming-Jen Chang, Tzu-Chien Fang, Hsing-Yang Tsai, Ming-Hui Luo, Kew-Yu Chen

**Affiliations:** a Department of Chemical Engineering, Feng Chia University, 40724 Taichung, Taiwan

## Abstract

In the title compound, C_10_H_5_N_3_O_2_, the benzyl­idene­malono­nitrile unit is nearly planar, with a maximum deviation of 0.129 (2) Å for a terminal N atom; the nitro group is approximately coplanar with the benzene ring [dihedral angle = 8.8 (3)°]. An intra­molecular C—H⋯N hydrogen bond stabilizes the mol­ecular conformation.

## Related literature

For the preparation of the title compound, see: Baheti *et al.* (2011 ▶). For the spectroscopy and applications of benzyl­idenemalononitrile derivatives, see: Cao *et al.* (2010 ▶); Ding & Zhao (2010 ▶); Elinson *et al.* (2010 ▶); Herbivo *et al.* (2010 ▶); Shigemitsu *et al.* (2011 ▶); Ye *et al.* (2010 ▶). For related structures, see: El Brahmi *et al.* (2011 ▶); Karthikeyan *et al.* (2011 ▶); Mehdi *et al.* (2010 ▶); Ouzidan *et al.* (2011 ▶); Raza *et al.* (2010 ▶).
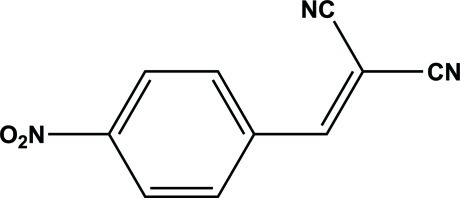


## Experimental

### 

#### Crystal data

C_10_H_5_N_3_O_2_
*M* *_r_* = 199.17
Orthorhombic, 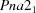

*a* = 19.5557 (9) Å
*b* = 3.8732 (2) Å
*c* = 11.9823 (5) Å
*V* = 907.58 (7) Å^3^
*Z* = 4
Cu *K*α radiation
μ = 0.89 mm^−1^
*T* = 297 K
0.76 × 0.60 × 0.18 mm

#### Data collection

Bruker SMART CCD area-detector diffractometer
Absorption correction: multi-scan (*SADABS*; Bruker, 2001 ▶) *T*_min_ = 0.674, *T*_max_ = 1.000
3111 measured reflections
1517 independent reflections
1420 reflections with *I* > 2σ(*I*)
*R*_int_ = 0.016

#### Refinement

*R*[*F* ^2^ > 2σ(*F* ^2^)] = 0.034
*wR*(*F* ^2^) = 0.091
*S* = 1.06
1517 reflections
136 parameters
1 restraint
H-atom parameters constrained
Δρ_max_ = 0.13 e Å^−3^
Δρ_min_ = −0.16 e Å^−3^
Absolute structure: Flack (1983 ▶), 582 Friedel pairs
Flack parameter: −0.2 (2)

### 

Data collection: *SMART* (Bruker, 2001 ▶); cell refinement: *SAINT* (Bruker, 2001 ▶); data reduction: *SAINT*; program(s) used to solve structure: *SHELXS97* (Sheldrick, 2008 ▶); program(s) used to refine structure: *SHELXL97* (Sheldrick, 2008 ▶); molecular graphics: *ORTEP-3 for Windows* (Farrugia, 1997 ▶); software used to prepare material for publication: *WinGX* (Farrugia, 1999 ▶).

## Supplementary Material

Crystal structure: contains datablock(s) I, global. DOI: 10.1107/S1600536812008896/xu5449sup1.cif

Structure factors: contains datablock(s) I. DOI: 10.1107/S1600536812008896/xu5449Isup2.hkl

Supplementary material file. DOI: 10.1107/S1600536812008896/xu5449Isup3.cml

Additional supplementary materials:  crystallographic information; 3D view; checkCIF report

## Figures and Tables

**Table 1:** Hydrogen-bond geometry (Å, °)

*D*—H⋯*A*	*D*—H	H⋯*A*	*D*⋯*A*	*D*—H⋯*A*
C1—H1*A*⋯N3	0.93	2.58	3.431 (3)	152

**Fig. 1.**
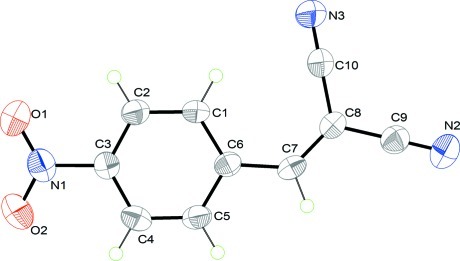
The molecular structure of the title compound, showing 50% probability displacement ellipsoids.

## supplementary crystallographic information

### Comment

Organic compounds bearing benzylidenemalononitrile moieties have attracted considerable attention due to their potential applications in the design of molecular devices (Cao *et al.*, 2010; Herbivo *et al.*, 2010; Shigemitsu *et al.*, 2011). In addition, the title compound and its derivatives have been used as potential precursors to prepare 5,7-diazaspiro[2,5]octane (Elinson *et al.*, 2010), 2-amino-4*H*-chromene-3-carbonitrile (Ding *et al.*, 2010) and 4*H*-pyran derivatives (Ye *et al.*, 2010).

The molecular structure of the title compound is shown in Figure 1. The nitro group is close to being coplanar with the benzene ring (dihedral angle = 8.8 (3)°), which is consistent with previous studies (El Brahmi *et al.*, 2011; Mehdi *et al.*, 2010; Ouzidan *et al.*, 2011; Raza *et al.*, 2010). In addition, the benzylidenemalononitrile moiety is nearly planar with a maximum deviation of 0.129 (2))Å for atom N2 (Karthikeyan *et al.*, 2011). An intramolecular C—H···N hydrogen bond stabilizes the molecular conformation.

### Experimental

The title compound was synthesized by the Knoevenagel condensation of malononitrile with 4-nitrobenzaldehyde (Baheti *et al.*, 2011). Colorless crystals suitable for the crystallographic studies reported here were isolated over a period of four weeks by slow evaporation from a chloroform solution.

### Refinement

H atoms were positioned geometrically (C—H = 0.93 Å) and allowed to ride on their parent atoms, with *U*_iso_(H) = 1.2*U*_eq_(C).

### Crystal data

**Table:**

C_10_H_5_N_3_O_2_	*F*(000) = 408
*M**_r_* = 199.17	*D*_x_ = 1.458 Mg m^−^^3^
Orthorhombic, *P**n**a*2_1_	Cu *K*α radiation, λ = 1.54178 Å
Hall symbol: P 2c -2n	Cell parameters from 2320 reflections
*a* = 19.5557 (9) Å	θ = 3.7–71.5°
*b* = 3.8732 (2) Å	µ = 0.89 mm^−^^1^
*c* = 11.9823 (5) Å	*T* = 297 K
*V* = 907.58 (7) Å^3^	Parallelepiped, colorless
*Z* = 4	0.76 × 0.60 × 0.18 mm

### Data collection

**Table:**

Bruker SMART CCD area-detector diffractometer	1517 independent reflections
Radiation source: fine-focus sealed tube	1420 reflections with *I* > 2σ(*I*)
Graphite monochromator	*R*_int_ = 0.016
ω scans	θ_max_ = 71.7°, θ_min_ = 4.5°
Absorption correction: multi-scan (*SADABS*; Bruker, 2001)	*h* = −24→15
*T*_min_ = 0.674, *T*_max_ = 1.000	*k* = −4→3
3111 measured reflections	*l* = −14→14

### Refinement

**Table:**

Refinement on *F*^2^	Secondary atom site location: difference Fourier map
Least-squares matrix: full	Hydrogen site location: inferred from neighbouring sites
*R*[*F*^2^ > 2σ(*F*^2^)] = 0.034	H-atom parameters constrained
*wR*(*F*^2^) = 0.091	*w* = 1/[σ^2^(*F*_o_^2^) + (0.0621*P*)^2^ + 0.0168*P*] where *P* = (*F*_o_^2^ + 2*F*_c_^2^)/3
*S* = 1.06	(Δ/σ)_max_ < 0.001
1517 reflections	Δρ_max_ = 0.13 e Å^−^^3^
136 parameters	Δρ_min_ = −0.16 e Å^−^^3^
1 restraint	Absolute structure: Flack (1983), 582 Friedel pairs
Primary atom site location: structure-invariant direct methods	Flack parameter: −0.2 (2)

### Special details

**Table:**

Geometry. All e.s.d.'s (except the e.s.d. in the dihedral angle between two l.s. planes) are estimated using the full covariance matrix. The cell e.s.d.'s are taken into account individually in the estimation of e.s.d.'s in distances, angles and torsion angles; correlations between e.s.d.'s in cell parameters are only used when they are defined by crystal symmetry. An approximate (isotropic) treatment of cell e.s.d.'s is used for estimating e.s.d.'s involving l.s. planes.
Refinement. Refinement of *F*^2^ against ALL reflections. The weighted *R*-factor *wR* and goodness of fit *S* are based on *F*^2^, conventional *R*-factors *R* are based on *F*, with *F* set to zero for negative *F*^2^. The threshold expression of *F*^2^ > σ(*F*^2^) is used only for calculating *R*-factors(gt) *etc*. and is not relevant to the choice of reflections for refinement. *R*-factors based on *F*^2^ are statistically about twice as large as those based on *F*, and *R*- factors based on ALL data will be even larger.

### Fractional atomic coordinates and isotropic or equivalent isotropic displacement parameters (Å^2^)

**Table:**

	*x*	*y*	*z*	*U*_iso_*/*U*_eq_	
O1	0.19540 (9)	1.1888 (6)	−0.10487 (14)	0.0798 (6)	
O2	0.17348 (9)	1.4626 (5)	0.04698 (17)	0.0790 (6)	
N1	0.21000 (8)	1.2840 (4)	−0.01057 (15)	0.0530 (4)	
N2	0.61988 (11)	0.5187 (6)	0.25478 (17)	0.0672 (5)	
N3	0.53572 (10)	0.5513 (7)	−0.07973 (17)	0.0743 (6)	
C1	0.38494 (10)	0.9176 (5)	0.00650 (14)	0.0445 (4)	
H1A	0.4165	0.8120	−0.0404	0.053*	
C2	0.32231 (10)	1.0153 (5)	−0.03437 (15)	0.0449 (4)	
H2A	0.3110	0.9738	−0.1086	0.054*	
C3	0.27635 (9)	1.1752 (5)	0.03563 (14)	0.0405 (4)	
C4	0.29078 (10)	1.2411 (5)	0.14638 (16)	0.0471 (5)	
H4A	0.2591	1.3505	0.1922	0.056*	
C5	0.35351 (10)	1.1396 (5)	0.18681 (15)	0.0447 (4)	
H5A	0.3641	1.1811	0.2613	0.054*	
C6	0.40158 (9)	0.9766 (4)	0.11918 (14)	0.0387 (4)	
C7	0.46578 (10)	0.8685 (5)	0.17120 (14)	0.0415 (4)	
H7A	0.4690	0.9154	0.2471	0.050*	
C8	0.52069 (10)	0.7129 (4)	0.12720 (15)	0.0408 (4)	
C9	0.57642 (11)	0.6105 (5)	0.19838 (16)	0.0478 (5)	
C10	0.52915 (9)	0.6242 (6)	0.01095 (17)	0.0489 (5)	

### Atomic displacement parameters (Å^2^)

**Table:**

	*U*^11^	*U*^22^	*U*^33^	*U*^12^	*U*^13^	*U*^23^
O1	0.0629 (11)	0.1147 (15)	0.0617 (10)	0.0171 (10)	−0.0179 (8)	−0.0051 (10)
O2	0.0585 (9)	0.0933 (14)	0.0852 (13)	0.0283 (9)	0.0017 (9)	−0.0088 (10)
N1	0.0435 (9)	0.0578 (10)	0.0577 (12)	0.0029 (7)	0.0036 (8)	0.0085 (9)
N2	0.0659 (12)	0.0835 (14)	0.0521 (10)	0.0141 (11)	−0.0141 (10)	0.0016 (9)
N3	0.0567 (11)	0.1197 (18)	0.0464 (10)	0.0190 (11)	−0.0019 (9)	−0.0203 (11)
C1	0.0467 (9)	0.0544 (11)	0.0324 (8)	0.0064 (8)	0.0011 (7)	−0.0025 (8)
C2	0.0470 (9)	0.0543 (11)	0.0334 (8)	0.0003 (8)	−0.0011 (8)	−0.0006 (8)
C3	0.0391 (8)	0.0424 (9)	0.0399 (10)	−0.0019 (7)	0.0009 (7)	0.0045 (7)
C4	0.0480 (10)	0.0506 (11)	0.0426 (10)	0.0023 (9)	0.0106 (8)	−0.0029 (9)
C5	0.0501 (10)	0.0529 (11)	0.0312 (8)	−0.0032 (8)	0.0027 (8)	−0.0009 (8)
C6	0.0447 (9)	0.0394 (9)	0.0321 (8)	−0.0029 (7)	0.0016 (7)	0.0030 (7)
C7	0.0515 (10)	0.0439 (11)	0.0290 (7)	−0.0038 (8)	−0.0023 (7)	0.0013 (7)
C8	0.0447 (9)	0.0407 (9)	0.0370 (9)	−0.0041 (8)	−0.0039 (7)	0.0024 (8)
C9	0.0502 (10)	0.0520 (11)	0.0413 (10)	0.0009 (9)	−0.0029 (9)	0.0011 (9)
C10	0.0401 (9)	0.0618 (12)	0.0449 (10)	0.0041 (8)	−0.0009 (8)	−0.0037 (9)

### Geometric parameters (Å, º)

**Table:**

O1—N1	1.222 (2)	C3—C4	1.381 (3)
O2—N1	1.210 (2)	C4—C5	1.376 (3)
N1—C3	1.472 (2)	C4—H4A	0.9300
N2—C9	1.143 (3)	C5—C6	1.393 (3)
N3—C10	1.130 (3)	C5—H5A	0.9300
C1—C2	1.372 (3)	C6—C7	1.463 (3)
C1—C6	1.407 (2)	C7—C8	1.339 (3)
C1—H1A	0.9300	C7—H7A	0.9300
C2—C3	1.377 (3)	C8—C10	1.444 (3)
C2—H2A	0.9300	C8—C9	1.440 (3)
			
O2—N1—O1	124.15 (19)	C4—C5—C6	121.76 (17)
O2—N1—C3	118.00 (17)	C4—C5—H5A	119.1
O1—N1—C3	117.85 (17)	C6—C5—H5A	119.1
C2—C1—C6	120.27 (17)	C5—C6—C1	118.41 (17)
C2—C1—H1A	119.9	C5—C6—C7	117.46 (17)
C6—C1—H1A	119.9	C1—C6—C7	124.11 (16)
C3—C2—C1	119.29 (17)	C8—C7—C6	130.47 (17)
C3—C2—H2A	120.4	C8—C7—H7A	114.8
C1—C2—H2A	120.4	C6—C7—H7A	114.8
C2—C3—C4	122.37 (17)	C7—C8—C10	125.34 (17)
C2—C3—N1	118.37 (15)	C7—C8—C9	119.85 (17)
C4—C3—N1	119.25 (16)	C10—C8—C9	114.78 (16)
C5—C4—C3	117.89 (18)	N2—C9—C8	177.8 (2)
C5—C4—H4A	121.1	N3—C10—C8	179.3 (3)
C3—C4—H4A	121.1		
			
C6—C1—C2—C3	0.8 (3)	C3—C4—C5—C6	0.2 (3)
C1—C2—C3—C4	−0.2 (3)	C4—C5—C6—C1	0.4 (3)
C1—C2—C3—N1	178.79 (16)	C4—C5—C6—C7	−177.94 (17)
O2—N1—C3—C2	−170.72 (19)	C2—C1—C6—C5	−0.9 (3)
O1—N1—C3—C2	9.0 (3)	C2—C1—C6—C7	177.29 (17)
O2—N1—C3—C4	8.3 (3)	C5—C6—C7—C8	−179.0 (2)
O1—N1—C3—C4	−172.0 (2)	C1—C6—C7—C8	2.8 (3)
C2—C3—C4—C5	−0.3 (3)	C6—C7—C8—C10	2.3 (3)
N1—C3—C4—C5	−179.30 (17)	C6—C7—C8—C9	−175.70 (17)

### Hydrogen-bond geometry (Å, º)

**Table:**

*D*—H···*A*	*D*—H	H···*A*	*D*···*A*	*D*—H···*A*
C1—H1*A*···N3	0.93	2.58	3.431 (3)	152

## References

[bb1] Baheti, A., Singh, P. & Thomas, K. R. J. (2011). *Dyes Pigm.* **88**, 195–203.

[bb2] Bruker (2001). *SMART*, *SAINT* and *SADABS* Bruker AXS Inc., Madison, Wisconsin, USA.

[bb3] Cao, X., Wen, Y., Guo, Y., Yu, G., Liu, Y. & Yang, L.-M. (2010). *Dyes Pigm.* **84**, 203–207.

[bb4] Ding, D. & Zhao, C.-G. (2010). *Tetrahedron Lett.* **51**, 1322–1325. 10.1016/j.tetlet.2009.12.139 PMC2821098 20161684

[bb5] El Brahmi, N., Benchidmi, M., Essassi, E. M., Ladeira, S. & Ng, S. W. (2011). *Acta Cryst.* E**67**, o3260. 10.1107/S1600536811046927 PMC3238920 22199769

[bb6] Elinson, M. N., Vereshchagin, A. N., Stepanov, N. O., Zaimovskaya, T. A., Merkulova, V. M. & Nikishin, G. I. (2010). *Tetrahedron Lett.* **51**, 428–431.

[bb7] Farrugia, L. J. (1997). *J. Appl. Cryst.* **30**, 565.

[bb8] Farrugia, L. J. (1999). *J. Appl. Cryst.* **32**, 837–838.

[bb9] Flack, H. D. (1983). *Acta Cryst.* A**39**, 876–881.

[bb10] Herbivo, C., Comel, A., Kirsch, G., Fonseca, A. M. C., Belsley, M. & Raposo, M. M. M. (2010). *Dyes Pigm.* **86**, 217–226.

[bb11] Karthikeyan, S., Sethusankar, K., Devaraj, A. & Bakthadoss, M. (2011). *Acta Cryst.* E**67**, o3469. 10.1107/S1600536811049816 PMC3239096 22199944

[bb12] Mehdi, S. H., Sulaiman, O., Ghalib, R. M., Yeap, C. S. & Fun, H.-K. (2010). *Acta Cryst.* E**66**, o1845. 10.1107/S1600536810024475 PMC3006776 21588044

[bb13] Ouzidan, Y., Kandri Rodi, Y., Saffon, N., Essassi, E. M. & Ng, S. W. (2011). *Acta Cryst.* E**67**, o558. 10.1107/S1600536811003503 PMC3052093 21522323

[bb14] Raza, A. R., Nisar, B. & Tahir, M. N. (2010). *Acta Cryst.* E**66**, o1852. 10.1107/S1600536810024621 PMC3007066 21588050

[bb15] Sheldrick, G. M. (2008). *Acta Cryst.* A**64**, 112–122. 10.1107/S0108767307043930 18156677

[bb16] Shigemitsu, Y., Wang, B.-C., Nishimura, Y. & Tominaga, Y. (2011). *Dyes Pigm.* **92**, 580–587.

[bb17] Ye, Z., Xu, R., Shao, X., Xu, X. & Li, Z. (2010). *Tetrahedron Lett.* **51**, 4991–4994.

